# Emerging insights into synapse dysregulation in Alzheimer’s disease

**DOI:** 10.1093/braincomms/fcac083

**Published:** 2022-04-08

**Authors:** Raquel Martínez-Serra, Lidia Alonso-Nanclares, Kwangwook Cho, K. Peter Giese

**Affiliations:** 1 Department of Basic and Clinical Neuroscience, Institute of Psychiatry, Psychology and Neuroscience, King’s College London, London SE5 9NU, UK; 2 Instituto Cajal (CSIC - Consejo Superior de Investigaciones Científicas), Avda. Doctor Arce 37, 28002 Madrid, Spain; 3 Laboratorio Cajal de Circuitos Corticales (CTB), Universidad Politécnica de Madrid, Campus de Montegancedo s/n, Pozuelo de Alarcón 28223, Madrid, Spain; 4 UK-Dementia Research Institute at King’s College London, London SE5 9NU, UK

**Keywords:** Alzheimer’s disease, three-dimensional electron microscopy, synapses, multi-spine bouton, multi-innervated spine

## Abstract

Alzheimer’s disease is the leading cause of dementia and a growing worldwide problem, with its incidence expected to increase in the coming years. Since synapse loss is a major pathology and is correlated with symptoms in Alzheimer’s disease, synapse dysfunction and loss may underlie pathophysiology. In this context, this review focuses on emerging insights into synaptic changes at the ultrastructural level. The three-dimensional electron microscopy technique unequivocally detects all types of synapses, including multi-synapses, which are indicators of synaptic connectivity between neurons. In recent years it has become feasible to perform sophisticated three-dimensional electron microscopy analyses on post-mortem human Alzheimer’s disease brain as tissue preservation and electron microscopy techniques have improved. This ultrastructural analysis found that synapse loss does not always precede neuronal loss, as long believed. For instance, in the transentorhinal cortex and area CA1 of the hippocampus, synapse loss does not precede neuronal loss. However, in the entorhinal cortex, synapse loss precedes neuronal loss. Moreover, the ultrastructural analysis provides details about synapse morphology. For example, changes in excitatory synapses’ post-synaptic densities, with fragmented postsynaptic densities increasing at the expense of perforated synapses, are seen in Alzheimer’s disease brain. Further, multi-synapses also appear to be altered in Alzheimer’s disease by doubling the abundance of multi-innervated spines in the transentorhinal cortex of Alzheimer’s disease brain. Collectively, these recent ultrastructural analyses highlight distinct synaptic phenotypes in different Alzheimer’s disease brain regions and broaden the understanding of synapse alterations, which may unravel some new therapeutic targets.

## Teaser

Martínez-Serra *et al*. report that synapse changes in post-mortem Alzheimer’s disease brain regions are heterogenous. Further, changes in multi synapses are most prominent in the disease, indicating that pre- and post-synapses degenerate independently and that a change in neuronal connectivity is relevant for cognitive decline in Alzheimer’s disease.

## Graphical Abstract

**Graphical Abstract:**
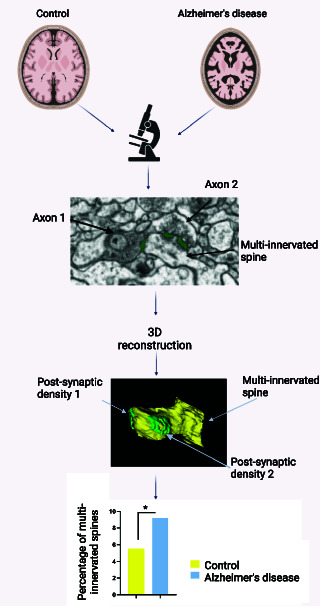

## Introduction

Alzheimer’s disease is the most common cause of dementia. Growing evidence suggests that memory impairment in Alzheimer’s disease correlates with synapse loss in the forebrain.^1–5^ For instance, synapse loss in the hippocampus, dentate gyrus, inferior temporal gyrus and superior frontal cortex negatively correlates with performance in various types of memory tasks.^6–10^ Given this correlation, it is important to understand how synapses are affected in Alzheimer’s disease in order to be able to intervene and reverse synaptic changes to possibly prevent and/or rescue cognitive and memory impairment.

To this date, several methods have been used to assess synapse density. For example, indirect quantification of pre- and post-synaptic proteins, such as synaptophysin, synapsin-1 and postsynaptic density protein 95 (PSD-95) by immunohistochemistry, ELISA, dot-blot and western blot.^4,9,11–15^ However, these methods estimate, at very best, the presence of specific synaptic proteins in the pre- or post-synaptic compartments, but they cannot provide the detailed context of pre- and post-synaptic architecture and determine the progression of pathology in the disease. For example, the presynaptic marker CSPalpha is reduced in Alzheimer’s disease before synaptophysin levels are affected,^16^ suggesting that at least some synaptic markers can have a reduction in expression without any impact on synapse numbers. Further, DeKosky and colleagues^4^ did not find a correlation between synaptophysin expression and cognitive function, even though synapse density correlated with cognitive abilities, showing the inaccuracy of relying on synaptic protein expression as markers for synapses.

It is also very common to assess synapse density and morphology by fluorescence imaging of dendritic spines. However, it should be noted that such imaging does not assure that the dendritic spines have a presynaptic input, and it can also not distinguish between multi-synapses and one-input-one-spine synapses, except for the recently developed super-resolution imaging with the DNA-paint method for cultured neurons.^17,18^ Further, subcellular components within the spines, such as the spine apparatus, cannot be identified with light microscopy in contrast with electron microscopy (EM).

**Figure 1:**
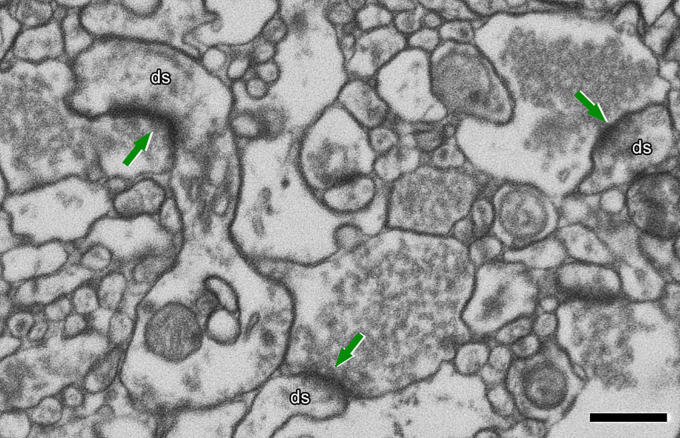
**Identification of synapses in EM image obtained by FIB/SEM on the transentorhinal cortex from post-mortem human brain (control).** Excitatory synapses (arrows) on dendritic spines (ds) are shown. Presynaptic elements contain numerous and visible vesicles. The arrows point at the asymmetric PSDs. Scale bar: 500 nm.

To study how synapses are changed in Alzheimer’s disease, it is important to use methods that allow for the unequivocal identification of synapses. EM is the gold standard for ultrastructure assay since it provides sufficient high resolution for a clear visualization of PSDs and presynaptic vesicles, making it possible to identify a synaptic connection on the nanometric scale (Fig. 1). EM also allows for the identification of synapses and their classification into asymmetric synapses (AS) and symmetric synapses (SS). This distinction is important as these two types of synapses correlate with different functions: AS are mostly glutamatergic and excitatory, while SS are mostly GABAergic and inhibitory.^19^

Serial sectioning TEM is a well-established technique to obtain three-dimensional data from ultrathin sections of brain tissue. However, obtaining a long series of ultrathin sections is extremely time-consuming, difficult and requires labour-intensive human interaction that prevents this approach from being widely employed (reviewed in^20^). However, the development of automated EM techniques represents an important advance. One of these techniques of three-dimensional (3D)-EM, called dual-beam microscopy, combines a high-resolution field-emission SEM column with a focused gallium ion beam (FIB), which permits the removal of thin layers of material from the sample surface on a nanometer scale. As soon as one layer of material is removed by the FIB, the exposed surface of the sample is imaged by the SEM using a backscattered electron detector. The sequential automated use of FIB milling and SEM imaging allows for obtaining long series of photographs of a 3D sample of selected brain regions (e.g. see reference ^21^). The FIB/SEM microscopy offers the advantage that the process of obtaining serial images is fully automated, eliminating the need for serial sectioning, the collection of ultrathin sections and the manual acquisition of microphotographs. Indeed, FIB/SEM is an excellent tool to study in detail the ultrastructure and alterations of the synaptic organization of the human brain, as shown by Blazquez- Blazquez-Llorca *et al*.^22^ who studied AD human tissue for the first time using this technique.

**Figure 2:**
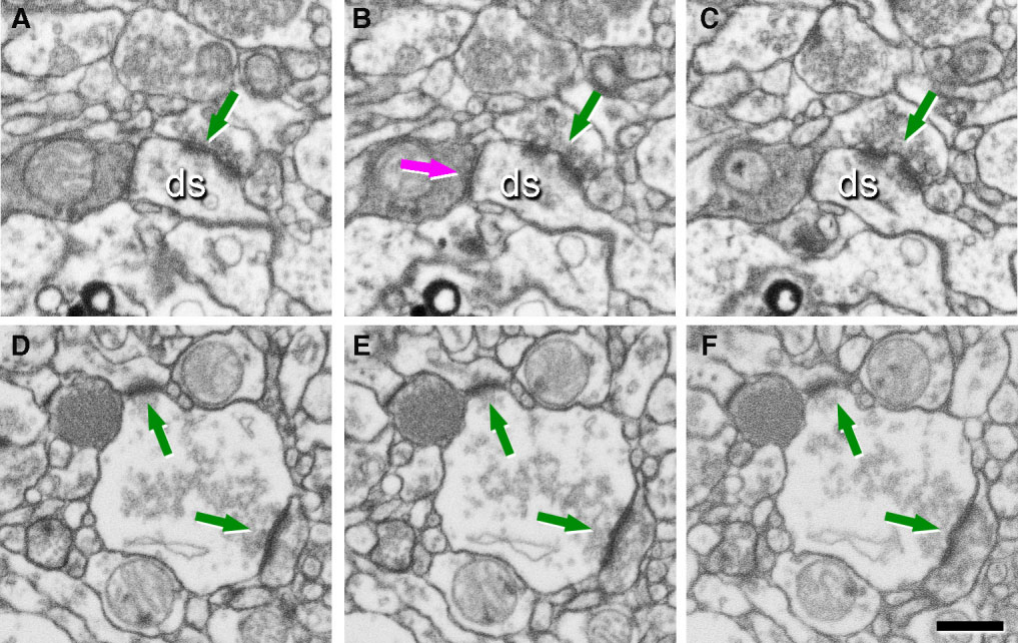
**Identification of synapses in EM serial images obtained by FIB/SEM on the transentorhinal cortex from post-mortem human brain (control).** (**A–C**) A sequence of serial images showing a multi-innervated dendritic spine (ds) with an excitatory **A–C** and an inhibitory synapse **B** indicated by arrows. (**D–F**) A sequence of serial images to illustrate a multi-synaptic bouton establishing two excitatory synapses with two dendritic spines (arrows). Scale bar in **F** 500 nm.

**Figure 3:**
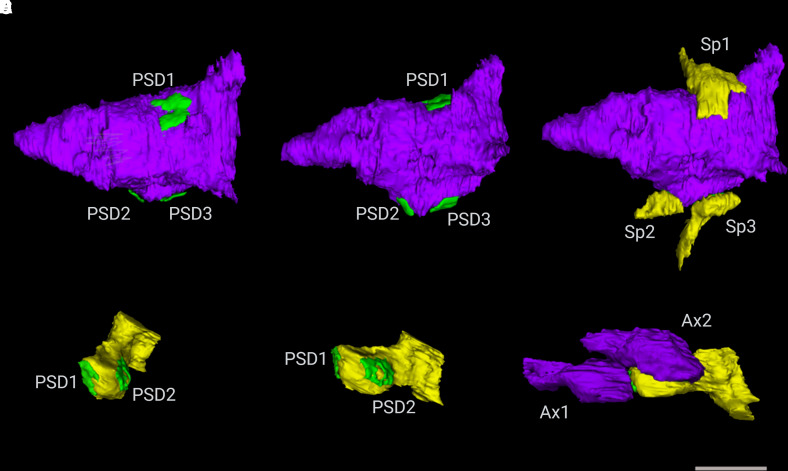
**Examples of 3D reconstructed axonal boutons establishing synapses with dendritic spines.** (**A**–**C**) 3D reconstructions of a multi-spine bouton after axis rotation of the axon. The MSB includes one axonal bouton, three post-synaptic densities (PSD) on **A–B** and three dendritic spines (Sp) on **C**. (**D**–**F**) 3D reconstructions of a multi-innervated spine after axis rotation of the dendritic spine. The MIS consists of one dendritic spine, two post-synaptic densities (PSD) on **D–E**, and two axonal boutons (Ax) on **F**. Scale bar (in F) indicates 1 μm in **A–F**.

Further, 3D EM is essential to identify synapses that have connections with multiple dendritic spines or with multiple presynaptic terminals, which can be considered as multi-output and multi-input, respectively (Fig. 2). Correspondingly, these synapse types are named multi-innervated spines (MIS) and multi-spine boutons (MSBs). 3D EM can identify and reconstruct the post-synaptic densities (PSDs) as independent elements, which can be achieved only by a 3D analysis at the EM level (Fig. 3) and will provide the ultrastructure synapse architecture in the brain.

Multi-synapses in Alzheimer’s disease have been overlooked for many years, but it is paramount to study these types of synapses as the presence and/or proportions of both types of multi-synapses change the connectivity between neurons and seem to contribute to learning and memory.^23^ For instance, Geinisman^24^ reported that trace eyeblink conditioning in rabbits increases MSB density in hippocampal CA1 stratum radiatum. Similarly, aged mice, as well as mutants with impaired long-term potentiation, are able to form and store hippocampal-dependent memories through the formation of MIS.^25–27^ Therefore, given the contribution of multi-synapses to cognition and memory, it is important to be able to identify and analyse these types of synapses in Alzheimer’s disease.

Despite providing resolution at the nanoscale level, to undoubtedly identify synapses, synapse types and subcellular structures, EM has some limitations. For instance, it is not possible to use EM imaging in living organisms; therefore, longitudinal studies to assess synaptic alteration during disease progression are not possible. Also, 3D-EM synapse reconstruction and analysis are very time-consuming.

Reviewed data come from FIB/SEM studies performed on human brain samples from control and AD cases (for details, see references ^28–32^). Briefly, brain tissue samples with a very short post-mortem delay (less than 4 h) were fixed in cold 4% paraformaldehyde. After fixation, the tissue was coronally sectioned. Serial sections were post-fixed and stained with uranyl acetate and then dehydrated and flat-embedded in Araldite.^33^ Embedded sections were glued onto a block. Blocks were glued onto a sample stub, and the top surface was coated with a layer of gold/palladium to facilitate charge dissipation. The blocks were used to obtain images stacks using a dual-beam microscope (FIB/SEM; Crossbeam® 540 electron microscope, Carl Zeiss NTS GmbH, Oberkochen, Germany). FIB/SEM images were obtained avoiding the neuronal and glial somata, blood vessels and also Aβ plaques in order to eliminate the effect of alterations of synapses in the vicinity of Aβ-plaques, which has been described previously (e.g. see^22^) FIBSEM images were analysed using EspINA software, which allows for the 3-dimensional reconstruction of synapses (Video 1).

In this review, we will focus on the emerging insights of synaptic changes in post-mortem Alzheimer’s disease brain derived from recent 3D EM analyses, which became feasible due to very short post-mortem delay to assure high tissue preservation. An example of a post-mortem EM image is presented in Fig. 1. This analysis provides the best knowledge to date about ultrastructural changes in synapses in Alzheimer’s disease, which is essential for an understanding of the mechanisms underlying synaptic degeneration in the disease. There is a notion that 3D EM analysis occasionally contradicts what was observed with traditional EM analysis, such as in the CA1 region of the hippocampus.^7^ However, it is mandatory that more brain regions are analysed using 3D EM before reaching any general conclusion.

## Does synapse loss precede neuronal depletion in post-mortem human Alzheimer’s disease brain?

An important question is whether synapse loss in Alzheimer’s disease is a cause or a consequence of neurodegeneration. The recent 3D EM analysis has revealed that there is no simple answer to this question. For instance, brain atrophy, which includes neuronal loss, is greater than 30% in the CA1 region of the hippocampus and the transentorhinal cortex in post-mortem human Alzheimer’s disease brain. However, synapse density is not reduced in the surviving tissue in the transentorhinal cortex and CA1 stratum pyramidale and stratum radiatum.^30,31^ In contrast, in layers II and III of the entorhinal cortex, synapse density is substantially reduced.^29^ Thus, in the transentorhinal cortex and in the CA1 region of the hippocampus, synapse loss appears to be associated with neuronal loss, whereas in the entorhinal cortex, synapse loss may precede neuronal loss.

Table 1 summarizes the relationship between synapse and neuronal loss in different post-mortem Alzheimer’s disease brain areas using 3D EM analysis. In most, but not all, analysed brain regions, synapse loss seems to be a consequence of neuronal death rather than the cause of neurodegeneration. The fact that this is not always the case, as in the analysed layers of the entorhinal cortex, suggests that synapse vulnerability may differ between brain areas and/or layers. For example, synapses in CA1 stratum pyramidale may be more resistant towards degeneration than synapses in layers II and III of the entorhinal cortex.

**Table 1:** Summary of neuron and synapse density changes in Alzheimer’s disease

	Neuron density	Synapse density	Synapse loss precedes neuron loss	References
CA1 stratum pyramidale (hippocampus)	↓	=	No	^31^
CA1 stratum radiatum (hippocampus)	↓	=	No	^31^
Layers 2 and 3 entorhinal cortex	=	↓	Yes	^29^
Layer 2 transentorhinal cortex	↓	=	No	^30^

↓Means a decrease observed at the ultrastructure level and = shows no change.

Region-specific differences in alterations of calcium/calmodulin-dependent kinase II (CaMKII) expression may contribute to this range of synapse vulnerability.^34^ αCaMKII, which is known to be involved in synaptic plasticity and memory formation^35^ is also a tau kinase.^36^ Strikingly, only CA1 pyramidal neurons in Alzheimer’s disease hippocampus have elevated αCaMKII expression.^34^ CA3 pyramidal neurons and granule cells in dentate gyrus have no altered αCaMKII expression, but the activity of αCaMKII at synapses is impaired, affecting the functioning of these neurons.^34^ These distinct αCaMKII changes correlate with substantial loss of CA1 pyramidal neurons, but almost no loss of CA3 pyramidal neurons nor granule cells in the dentate gyrus of severe Alzheimer’s disease hippocampus.^37^

## Which synapses are altered or missing in post-mortem human Alzheimer’s disease brain?

It is of interest whether in Alzheimer’s disease brain particular synapse types are more prone to degeneration. Recent 3D EM analysis suggests that the ratio of excitatory towards inhibitory synapses is not altered in the transentorhinal cortex or in CA1 stratum pyramidale and stratum radiatum, where also no synapse loss on surviving neurons seems to occur.^30,31^ This ratio also remains unchanged in the entorhinal cortex in Alzheimer’s disease, despite synapse loss on surviving neurons.^29,38^ These findings suggest that both excitatory and inhibitory synapses are equally vulnerable in Alzheimer’s disease. However, due to the limited number of brain regions investigated, we cannot rule out that Alzheimer’s disease has a different impact on excitatory and inhibitory synapses in other brain regions.

Morphology of PSDs, which can be either macular or non-macular (horseshoe-shaped, perforated and fragmented)^39,40^ also seems to be altered in Alzheimer’s disease brain. Different alterations are detected in distinct brain areas, but some common traits can be observed. For instance, the morphology of inhibitory synapses does not seem to be modified in any of the analysed brain regions. As for excitatory synapses, those with fragmented PSDs are generally increased, while perforated synapses are more often decreased in Alzheimer’s disease.^28,29,31^

Synaptic connections can target different parts of the post-synaptic cell, which may have mechanistic implications. For instance, synapses can be seen in dendritic spines, further divided into spine heads or necks, and also in dendritic shafts. Considering these parameters, the synaptic location appears to be altered in Alzheimer’s disease, but only in brain regions where synapse loss does not precede neuronal loss, i.e. hippocampus and transentorhinal cortex.^28,29,31^ In these brain areas, the number of axonal boutons targeting dendritic spines is reduced, while excitatory synapses on dendritic shafts are increased.^28,31^ Inhibitory axodendritic synapses are reduced in CA1 stratum radiatum.^31^

In summary, synapse morphology and location are altered in Alzheimer’s disease (Table 2), but more research is needed to establish whether this is a general feature and/or if particular synapse types are predisposed to degeneration in Alzheimer’s disease. Especially research on brain regions where synapse loss precedes neuronal death in Alzheimer’s disease is essential because here, just one brain region with this characteristic has been considered, and this is not enough to reach a meaningful conclusion.

**Table 2:** Summary of synapse subtype changes in Alzheimer’s disease

	AS:SS	Synapse morphology (AS)	Synapse targeting	References
CA1 stratum pyramidale (hippocampus)	=	↓ perforated	↓ axospinous AS, ↑ axodendritic AS	^31^
CA1 stratum radiatum (hippocampus)	=	**=**	↓ axodendritic SS	^31^
Layers 2 and 3 entorhinal cortex	=	**Layer 2**: ↑ horseshoe-shaped **Layer 3**: ↑ fragmented and macular, ↓ perforated	=	^29^
Layer 2 transentorhinal cortex	=	↑ fragmented	↓ axospinous AS, ↑ axodendritic AS	^28,30^

↓ and ↑indicate changes observed at the ultrastructural levels,  = means no change. AS, asymmetric synapses; SS, symmetric synapses.

## What are the features of surviving synapses in late-stage Alzheimer’s disease brain?

Recent studies have also tested for possible differences in synaptic enlargement in relation to synapse type and PSD morphology rather than a general enlargement of synapses. In the entorhinal cortex, only excitatory synapses in layer II, and perforated excitatory synapses in layer III, are enlarged.^29^ However, enlarged synapses do not occur in the transentorhinal cortex or any hippocampal CA1 layer.^28,31^

Previously, the generally accepted idea was that a reduction in synapse number correlates with a significant increase in synapse size in the post-mortem Alzheimer’s disease brain.^4,10,41–43^ This previous work illustrated an increase of the PSD size and apposition length or synaptic apposition surface by about 25% in Alzheimer’s disease compared with age-matched control subjects.^7,42–45^ As a result of this correlation, a common inference was that the total synaptic contact area was maintained to a similar level than in controls.^10,42,43^ However, in contrast with this previous work, a general synaptic enlargement is not detected with recent 3D EM analysis in human samples from Alzheimer’s disease brains (Table 3), indicating that the total synaptic contact area may also be lost in Alzheimer’s disease.

**Table 3:** Summary of synapse size change in Alzheimer’s disease

	Synapse enlargement	Maintenance of total synaptic contact area	References
CA1 stratum pyramidale (hippocampus)	No	?	^31^
CA1 stratumradiatum (hippocampus)	No	?	^31^
Layers 2 and 3 entorhinal cortex	Only excitatory synapses in layer 2 and excitatory perforated synapses in layer 3	Maybe	^29^
Layer 2 transentorhinal cortex	No	?	^28^

## Does Alzheimer’s disease alter synapse connectivity?

**Figure 4:**
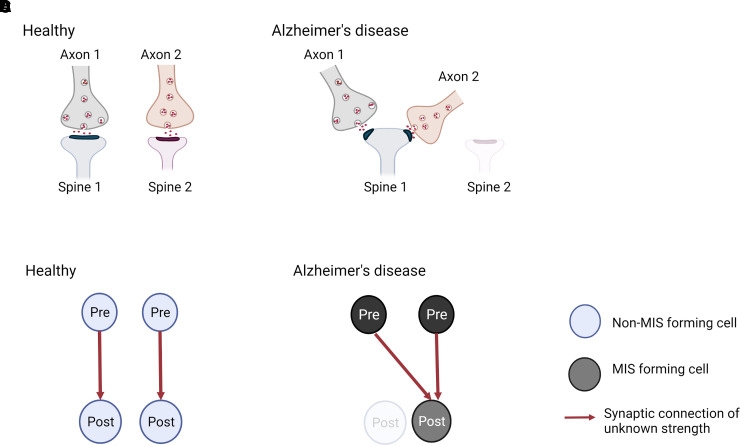
**Model for generation of multi-innervated dendritic spines (MIS) in Alzheimer’s disease brains and impact on synaptic connectivity.** (**A** and **B**) Two synapses in a healthy brain and Alzheimer’s disease brain are shown. (**A**) In a healthy brain, one synapse is formed between axon 1 and spine 1, while the other synapse is made between axon 2 and spine 2. (**B**) In Alzheimer’s disease, spine 2 has degenerated, and its presynaptic input from axon 2 established a new connection with spine 1, generating an MIS. As a consequence, synapse density is maintained, but MIS number is increased. (**C** and **D**) Illustration of the difference in synaptic connectivity and resulting information flow as a consequence of dendritic spine loss and MIS generation in Alzheimer’s disease. It is less likely that the higher MIS number increases connectivity between two neurons (scenario not shown), as two axonal branches from one pre-synaptic neuron would have to connect to one spine. - Created with BioRender.

The remaining question is whether synapse alterations in Alzheimer’s disease affect connectivity between pre- and post-synaptic neurons. It is known that synaptic connections can be clustered into MSBs and MIS (Fig. 2).^46,47^ Alterations in MSB and MIS numbers and complexity change brain connectivity and are thought to contribute to memory.^23^ For instance, the number of MSBs is increased in rabbit hippocampal CA1 stratum radiatum after eyeblink conditioning.^24^ Further, MISs have been suggested to be the mechanism responsible for hippocampal memory storage in aged mice as well as in LTP-impaired mouse.^25–27^ Despite the evidence linking MSB and MIS with memory, research on these types of synapses has been overlooked for many years, and they have not been considered much in studies of Alzheimer’s disease. Recently, however, MISs have been investigated in post-mortem Alzheimer’s disease brains. They are not altered in the CA1 region of the hippocampus, but they are doubled in the transentorhinal cortex of the Alzheimer’s disease brain.^28,31^ It is possible that in Alzheimer’s disease, some post-synaptic terminals become dysfunctional, degenerate or just disconnect from presynapses across a synapse, and then these presynaptic terminals would get a signal to find another existing spine to establish a new synapse, inducing the formation of MIS, and explaining the lack of synapse loss. Alternatively, new axonal terminals may be formed and incorporated into existing SSB, these becoming an MIS (Fig. 4).

**Figure 5:**
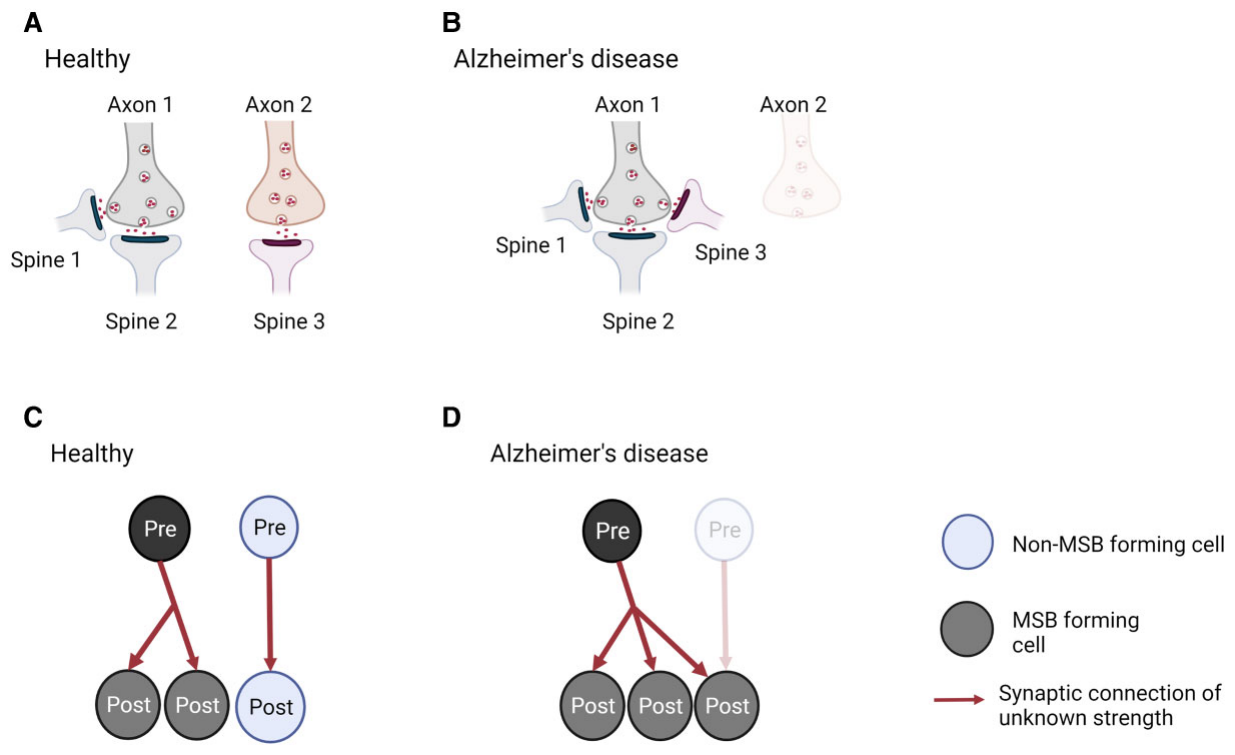
**Model for generation of multi-spine boutons in Alzheimer’s disease brains and impact on synaptic connectivity.** (**A** and **B**) Three synapses in a healthy and Alzheimer’s disease brain are shown. (**A**) An MSB is formed between axon 1 and spines 1 and 2, and a single synapse between axon 2 and spine 3 is shown for a healthy brain. (**B**) In Alzheimer’s disease, the presynaptic input from axon 2 has degenerated, and spine 3 from the single synapse established a new connection with the existing MSB. As a consequence, synapse density and MSB number are maintained, but MSB’s complexity is increased. (**C** and **D**) Illustration of the difference in synaptic connectivity and resulting information flow as a consequence of dendritic spine loss and MIS generation in Alzheimer’s disease. Note that in a healthy brain, the vast majority of most MSBs are formed between one presynaptic neuron and two post-synaptic neurons. Thus, the higher MSB complexity in Alzheimer’s disease brain is unlikely to include spines from the same dendrite, which would not lead to connecting of previously unconnected neurons (scenario not shown). Created with BioRender.

Further, our lab has found that the number of MSBs in the transentorhinal cortex, entorhinal cortex and stratum pyramidale superior of Alzheimer’s disease brains is similar to control levels (unpublished data). We hypothesize that though some spine-forming synapses in this region may become dysfunctional, degenerate, or disconnect from axons across the synapse, other spines that may have lost their presynaptic partners would get a signal to find another existing axonal bouton to establish a new synapse and explain the lack of synapse loss, the maintenance of the proportion of MSBs and maybe increase the number of connections per bouton. An alternative explanation is that new spines may be formed and incorporated into existing boutons to form new MSBs (Fig. 5).

Unfortunately, it is not possible to use 3D EM to investigate whether these alterations in MIS and MSB are caused by particular terminals degenerating or if they are newly formed since longitudinal studies are not possible. Either way, it is mandatory to investigate whether these new connections forming MIS or complex MSBs originate from the same neuron, hence increasing the connectivity in the brain, or if they arise from different axons or dendrites and therefore connect more cells. If the latter is true, and the connected cells have unrelated activity, memories encoded at different synapses might be ‘mixed’, possibly affecting memory and cognition.

Given the implications that changes in MIS and/or MSB appear to have in brain connectivity and cognition, it should be investigated whether MIS and MSB alterations are seen throughout the Alzheimer’s disease brain or just in the transentorhinal cortex. Further, the functional impact of such changes in synaptic connectivity needs to be analysed in model systems.

## Concluding remarks and outstanding questions

Despite all research looking at synaptic alterations in Alzheimer’s disease, many outstanding questions remain to be addressed. With the development of newer and more advanced techniques, such as 3D EM and super-resolution imaging, along with the possibility of obtaining post-mortem brain tissue with minimal post-mortem delay and ensuring better tissue preservation, more detailed analyses can be carried out. Detailed synapse analysis is feasible, for instance, looking at specific synapse types (excitatory and inhibitory, macular and non-macular, etc), the location of the synapse within the post-synaptic cell (spine head, neck or dendritic shaft) as well as the quantification of multi-synapses (MIS and MSB). Therefore, recent studies using this technique have overcome some of the previous limitations and will provide a better and more accurate understanding of the disease pathology.

Regarding synapse and neuronal loss, it seems there is no homogeneity throughout the post-mortem Alzheimer’s disease brain, with synapse loss being associated with neuronal death in some but not all brain regions.^29–31^ This suggests different levels of resilience against synaptic dysfunction and degeneration between brain subregions. In order to target synaptic pathology in the most vulnerable regions, we need to understand what causes synapses to be more resistant towards dysfunction and degeneration in these areas. Excitatory and inhibitory synapses seem to be equally lost in Alzheimer’s disease, but more studies in other brain regions are needed in order to find whether this is a general principle in Alzheimer’s disease.

There is also no uniformity regarding changes in PSD morphology and synapse location within the post-synaptic cell in Alzheimer’s disease brains. These alterations appear to happen in specific neurons, with excitatory neurons being more affected.^28,29,31^ Changes in PSD morphology and synapse location could affect synapse function, maybe altering the excitatory-inhibitory balance, and affecting the cellular mechanism of learning and memory. Therefore, it is important to investigate why these particular synapses are altered and the implications of these changes.

Notably, 3D EM studies did not detect any synapse enlargement in most areas of the Alzheimer’s disease brain, in contrast to what has been reported before.^10,41–44^ Therefore, the maintenance of the total synaptic contact area also appears not to be a general feature of Alzheimer’s disease. However, these studies looked at neuropil synapses; therefore, a compensatory enlargement of perisomatic synapses cannot be ruled out. Unfortunately, studies using 3D EM to analyse post-mortem Alzheimer’s disease brains are still scarce, and this makes it difficult to know if synaptic enlargement may be a feature of synapses on cell bodies and/or in other brain regions. It is also important to know why in the transentorhinal cortex specific neuropil synapses and not others get bigger, what mechanisms underlie the size alteration and if these enlarged synapses are conserved, maybe in an attempt to store memories, or per contra, if they are in the process of dying.

Finally, MIS and MSBs also seem to be altered in Alzheimer’s disease. The reported changes could be part of a compensatory mechanism, trying to regain brain connectivity, leading to the disruption of stored memories. However, more 3D-EM analyses should be done to investigate whether these are common alterations in different brain areas and also if they represent an increased connectivity between the same neurons or a higher connectivity between different cells.

Despite the detailed ultrastructural analyses that 3D EM can offer, many questions remain unanswered. It is not yet clear why resilience towards synapse degeneration appears to be higher in some brain regions or why PSD morphology and synapse location are more commonly altered in excitatory synapses. Further, the role of abnormally enlarged neuropil synapses in the transentorhinal cortex and whether their formation should be enhanced or prevented or how this can be done also remains unknown. In addition, MIS and MSBs appear to be vulnerable in the transentorhinal cortex, but it still is unclear what their exact role is and whether they are important for memory storage or retrieval.

In order to tackle these questions, more studies are still needed in more brain regions to further investigate synaptic changes. Analyses of post-mortem brain tissue at the early stages of Alzheimer’s disease will also be necessary to discern between primary and secondary changes in association with pathology. Further, while 3D-EM is the gold standard for synapse identification, molecular mechanisms cannot be very well studied using this technique, as it involves immuno-EM, a laborious procedure for which it may be difficult to find suitable antibodies. However, single-molecule imaging techniques, such as super resolution microscopy (SRM), stochastic optical reconstruction microscopy (STORM) or DNA paint, are more suitable to study molecular mechanisms.^48^ On the other hand, these techniques cannot be used to identify multiple synapses, and they are not very well established for *in vivo* or *in situ* work. Therefore, findings using 3D-EM could be translated and looked at with a single-molecule imaging technique in order to unravel the molecular mechanisms underlying synaptic changes. For instance, findings using array tomography suggest that synapse density is decreased in proximity to amyloid oligomers surrounding plaques, and it increases in an approximately linear manner to reach similar levels as controls at 50 µm from a plaque.^49^ Similarly, FIB/SEM imaging has shown that the closer to an amyloid plaque, the smaller the number of synapses.^22^ Therefore, in the future, these techniques could be combined to investigate synaptic changes within 50 µm from an amyloid plaque and also to study the effects of hyperphosphorylated tau and neurofibrillary tangles in synapses. This can aid in finding the most accurate readout of pathophysiology to identify and study molecular and mechanistic processes happening in Alzheimer’s disease, which will deepen the understanding of disease-associated pathology and facilitate the development of new therapeutic targets.

## Data availability

Data sharing is not applicable to this article as no new data were created or analysed in this study.

## Abbreviations

3D-EM =: three-dimensional electron microscopy
AS =: asymmetric synapses
CaMKII =: calcium/calmodulin-dependent kinase II
EM =: electron microscopy
MIS =: multi-innervated spines
MSB =: multi-synaptic boutons
PSD =: postsynaptic density
PSD-95 =: postsynaptic density protein 95
SRM =: super-resolution microscopy
SS =: symmetric synapses
STORM =: stochastic optical reconstruction microscopy
